# Critical Leaf Magnesium Thresholds and the Impact of Magnesium on Plant Growth and Photo-Oxidative Defense: A Systematic Review and Meta-Analysis From 70 Years of Research

**DOI:** 10.3389/fpls.2019.00766

**Published:** 2019-06-18

**Authors:** Melanie Hauer-Jákli, Merle Tränkner

**Affiliations:** Department of Crop Sciences, Institute of Applied Plant Nutrition, Georg-August University Goettingen, Göttingen, Germany

**Keywords:** antioxidant enzyme, assimilation, critical concentration, reactive oxygen species, shoot root ratio, biomass, Mg deficiency

## Abstract

Magnesium (Mg) deficiency in plants is a widespread problem affecting productivity and quality in agricultural systems and forestry. Although numerous studies addressed the effect of Mg deficiency on biomass and photosynthetic CO_2_ assimilation, a summary evaluation of the effect of Mg supply on plant growth and photosynthesis is so far missing. We performed a systematic review and meta-analysis to collect and combine all relevant scientifically published data on the relationship between Mg nutrition and parameters that can be related to plant growth such as root and shoot biomass, harvestable yield, net CO_2_ assimilation and antioxidant enzyme activities. Moreover, this data pool was used to calculate critical Mg leaf concentrations for biomass and net CO_2_ assimilation for various plant species. Summarizing all studies included in our analysis, adequate Mg supply enhances net CO_2_ assimilation by 140%, leading to a biomass increase of 61% compared to Mg deficient control plants. Biomass partitioning between shoot and root is not only sensitive to Mg nutrition, but highly affected by the experimental cultivation technique. If plants are grown under adequate Mg supply during initial growth stages before exposing them to Mg deficiency, the shoot-root ratio was not affected. Otherwise, the shoot-root ratio significantly decreased in contrast to Mg deficient control plants. Concentration of reactive oxygen species decreased under adequate Mg supply by 31% compared to Mg deficient plants, resulting in decreased activities of most antioxidant enzymes and metabolites under adequate Mg supply. We combined all published data relating leaf Mg concentrations to growth and found a critical leaf Mg range for dry weight between 0.1 and 0.2% which was valid for numerous crop species such as wheat, potato, rice, maize, sorghum and barley. Critical leaf Mg concentrations for net CO_2_ assimilation were higher than for biomass for most species, e.g., potato, rice, citrus, and cotton. In conclusion, our evaluation can be used to identify Mg nutritional status in plants and may help to optimize fertilization strategies. It quantifies the demand of Mg for various crop and tree species for maintaining important physiological processes such as net CO_2_ assimilation that is required for optimal plant growth and yield.

## Introduction

Since pioneering work on mineral plant nutrition in the late nineteenth and early twentieth century, our fundamental understanding of the complex interactions between plant nutritional status and parameters of growth, development, and physiology has been continuously growing. Plant production appeared to be primarily limited by nitrogen, phosphorus, and potassium, and research was focused on these mineral nutrients. Magnesium (Mg) seemed to be neglected and was even termed “a forgotten element in crop production” (Cakmak and Yazici, 2010). This is reflected by the chronology of publications used in this meta-analysis: within a 50 year period, from 1960 to 2009, 45 relevant studies were published whereas 61 studies were published within the last 8 years from 2010 to 2018.

In recent years, the effect of Mg on plant shoot and root formation, photosynthetic performance, and cellular stress defense mechanisms was targeted in a growing number of scientific studies on various crops and plant species (e.g., Tang et al., 2012; Blasco et al., 2015; Huang et al., 2016; Tränkner et al., 2016; da Silva et al., 2017; Yang et al., 2017; Rehman et al., 2018). Comprehensive reviews on the role of Mg in plant physiology (Guo et al., 2016; Chen Z. C. et al., 2018) and specifically in photosynthetic processes (Tränkner et al., 2018) were published. Magnesium has a dominant role in photosynthesis and associated processes in the chloroplast, where up to 35% of leaf Mg is located (Cakmak and Yazici, 2010). The chloroplast ultrastructure, i.e., grana formation, requires Mg. The divalent Mg cation screens negative surface charges on the thylakoid membranes and thus, allows the thylakoid membranes to stack (Puthiyaveetil et al., 2017). Magnesium is required for the synthesis of chlorophyll (Masuda, 2008) and is involved in both Ribulose-1,5-bisphosphat-carboxylase/-oxygenase (Rubisco) activation by forming a complex with Rubisco activase, and for Rubisco activity by binding to the carbamate group of Rubisco (Portis, 2003). As a direct (Rubisco activation/activity) or indirect (chloroplast ultrastructure, chlorophyll synthesis) consequence of Mg functioning, plants respond to Mg deficiency with a substantial reduction of net CO_2_ assimilation rates. As a result, an insufficient supply of Mg reduces rates of biomass formation. In this context, rates of CO_2_ assimilation were reported to decrease significantly in response to deficient Mg supply in a variety of crops such as maize (Jezek et al., 2015), sunflower (Lasa et al., 2000), sugar beet (Terry and Ulrich, 1974), spinach (Ze et al., 2009a), as well as in trees such as *Pinus* (Laing et al., 2000; Sun et al., 2001) and citrus (Tang et al., 2012; Yang et al., 2012). Photosynthetic limitation results in reduced capacity for biochemical utilization of absorbed light energy, inducing the formation of reactive oxygen species (ROS) such as superoxide radicals and hydrogen peroxide (H_2_O_2_). As ROS are highly toxic and can lethally damage cell components, the activity of anti-oxidant enzymes such as ascorbate peroxidase (APX) and superoxide dismutase (SOD) is expected to be upregulated in order to detoxify ROS to non-critical concentrations. However, reported results on the effects of Mg supply on ROS concentrations and corresponding enzyme activities often appear non-uniform or even contradictory. For instance, increased levels of ROS and anti-oxidant enzyme activities are reported for Mg deficient mulberry (Kumar Tewari et al., 2006) and *Capsicum annuum* (Riga et al., 2005). However, Ze et al. (2009b) reported increased levels of ROS in Mg deficient spinach together with a concordant drop in stress resistance (i.e., reduced activity of anti-oxidative enzymes such as SOD). Similarly, Rehman et al. (2018) reported higher enzyme activities of SOD and CAT, after plant foliage was sprayed with Mg.

Contrasting results are also reported in studies on carbon partitioning in Mg deficient plants. Very early symptoms of Mg deficiency are assigned to impairments in the development of sink organs such as roots because of impaired phloem export of photo-assimilates from highly productive source organs. The consequence is an impaired root growth—relative to shoots—that results in an increased shoot-root ratio under Mg deficiency as reported by some authors (Cakmak et al., 1994; Fischer et al., 1998; Kumar Tewari et al., 2006; Mengutay et al., 2013; da Silva et al., 2014; Chen C. T. et al., 2018). However, others report no effect of Mg deficiency on shoot-root ratios (Troyanos et al., 1997; Kumar Tewari et al., 2004; Verbruggen and Hermans, 2013; Tränkner et al., 2016). Some of them attribute this missing effect to a specific experimental setup: experimental plants are often supplied with sufficient amounts of Mg during the initial growth phase (in order to generate more biomass for experimental purpose) before exposing them to Mg deficiency. This specific cultivation technique may result in no or less pronounced changes in biomass partitioning as compared to plants cultivated under permanent Mg deficiency (Tränkner et al., 2016). So far, this hypothesis is only speculation as valid statistical evidence is missing.

In order to avoid situations of field scale Mg deficiency, precise knowledge of critical threshold values for Mg concentrations in the plant tissue is required. Different concepts of critical nutrient concentrations have been described such as the concept of Critical Nutrient Concentration by Ulrich (1952), the concept of Critical Nutrient Range by Dow and Roberts (1982), and a concept of Jones (1967) which is based on visual judgment of plant appearance. A critical leaf nutrient threshold is defined as the nutrient concentration at which a 5–10% loss of a specific plant growth factor occurs (Ulrich and Hills, 1967). Loneragan (1968) suggested distinguishing between the minimal concentration of a nutrient for growth and the functional nutrient requirement which is the minimal nutrient concentration to sustain metabolic functions without limiting growth. For many plant species, neither critical Mg concentrations for growth nor functional Mg requirements are available in the literature—among them major crops such as barley and potato. Additionally, progress in plant breeding is likely to alter critical tissue concentrations over time, and genotypic differences might occur, as for example those examined by Clárk (1983) and Labate et al. (2018). A recent compendium of critical Mg tissue concentrations for growth and photosynthesis covering various species so far does not exist. Moreover, studies that relate Mg concentrations to growth parameters of specific species are a possible source of determining critical values in cases were those are so far missing.

Hence, a systematic review and meta-analysis was performed to quantify the effect of Mg deficiency on photosynthetic performance as well as plant growth. The respective studies were used to calculate critical Mg leaf concentrations for biomass and net CO_2_ assimilation. Published data were collected on the relationship between Mg nutrition and parameters that are related to plant growth and stress resistance, i.e., root and shoot biomass, harvestable yield, net CO_2_ assimilation, and anti-oxidative enzyme activity. Critical Mg concentrations for crop quality traits were not separately investigated because Gerendás and Führs (2013) in their review clearly proved that Mg doses beyond the requirement for maximum yield do not have beneficial effects on crop quality of numerous investigated species.

In our meta-analysis, we combined data of 80 reports on the impact of Mg nutrition on plant growth and photosynthesis, and of 22 reports on its impact on photo-oxidative stress response. Further 28 reports were used for calculating species-specific critical leaf concentrations. The three major objectives were: (1) to provide estimates of the magnitude and significance of Mg fertilization on biomass formation, photosynthetic CO_2_ assimilation rates, and anti-oxidative enzyme activities; (2) to determine factors influencing the magnitude of these Mg responses; and (3) to calculate leaf Mg concentrations critical for plant growth and net CO_2_ assimilation under consideration of all relevant scientifically published data.

## Materials and Methods

### Search Strategy and Selection Criteria

A systematic literature search was conducted to collect all relevant published data related to the effect of Mg on plant growth and photosynthesis (timespan: 1945 to 2018). The following literature search using “advanced search” in ISI web of knowledge (www.webofknowledge.com) was performed on 12th of June 2018 and produced 1,374 hits (updated on 21st of September 2018 with 1,403 hits):

TS = [magnesium AND (photosynthesis OR assimilation OR yield OR biomass OR productivity) AND (fertilization OR deficiency OR nutrition)].

After title and abstract screening, 146 studies were downloaded and 80 studies were considered suitable for further analysis (Table 1). Studies included in the analysis contained individual comparisons of the parameters plant biomass, net CO_2_ assimilation, dry matter, fresh biomass, yield or leaf, shoot or whole plant Mg concentration, respectively, in relation to different levels of magnesium nutrition (Supplementary Materials 1, 2).

**Table 1:** Overview of calculated effects in the meta-analysis including number of studies and number of species per investigated parameter.

**Parameter**	**Number of studies**	**Number of species**	**Number of calculated effects**
Biomass total	41	32	89
Biomass leaf	12	9	36
Biomass root	36	27	75
Biomass shoot	38	30	118
Shoot-root ratio	33	26	78
Net CO_2_ assimilation (A_N_)	23^†^	15	46
Leaf Mg concentration	31^‡^	19	72
Reactive oxygen species (ROS)	12	10	20
ROS scavenging enzymes	19	15	137
ROS scavenging metabolites	15	12	69

† *A_N_ was measured either on fully expanded leaves (18 studies), separately in leaves of different ages (1 study) or on unspecified leaves (4 studies)*.

‡ *Leaf Mg concentration was measured either in fully expanded leaves (15 studies), separately in leaves of different ages (6 studies), total leaf (5 studies) or unspecified leaves (5 studies)*.

A second literature search was conducted to systematically review all available data about the effect of Mg nutrition on reactive oxygen species (ROS) and on enzymes (APX, Ascorbate Peroxidase; CAT, Catalase; DHAR, Dehydroascorbate reductase; GR, Glutathione reductase; POD, Peroxidase; SOD, Superoxide Dismutase) and metabolites (ASC, Ascorbate; DHA, Dehydroascorbate; GSH, reduced Glutathione; GSSG, oxidized Glutathione; MDA, Malondialdehyde) that are directly associated with the scavenging of ROS in plants. The following literature search using “advanced search” in ISI web of knowledge (www.webofknowledge.com) which was performed on 21st of June 2018 produced 61 hits (updated on 21st of September 2018 with 62 hits):

TS = [(H2O2 OR ROS OR “reactive oxygen” OR photooxidative) AND (“magnesium deficiency” OR “Magnesium stress” OR “Mg stress” OR “magnesium nutrition”)].

After title and abstract screening, 35 studies were downloaded from which 22 were used for further analysis.

An additional literature search was performed in Google Scholar to complete the data set that was used for the calculation of critical values and for the search of studies that already included an evaluation of critical Mg concentrations for growth. The critical concentration hereby is defined as the leaf or shoot Mg concentration at which a 10% loss of maximum yield, biomass or net CO_2_ assimilation occurs. A prerequisite for such a calculation is a curvilinear relationship between Mg concentration and parameters related to growth or photosynthesis. In total, 28 reports were used for critical value calculation. An additional 19 reports with already published critical Mg levels for certain species were included.

### Data Extraction

Mean data reported in the studies were categorized into “treatment” (different levels of Mg supply) and the comparison group “control” (no or clearly deficient Mg supply). The respective means, errors and sample sizes were compiled for each parameter (biomass, net CO_2_ assimilation, ROS production etc.) to compute effect sizes that were further used to statistically examine all studies in one analysis . In cases where the sample size (*n*) was missing, *n* = 3 was assumed. In cases where the given standard error or deviation was zero, a very small number was assumed to facilitate further calculations. Studies without standard deviation or standard error were excluded from the analysis except for critical value calculation.

Studies that compared parameters of Mg deficiency in temporal progression were included, but only the latest comparison with the longest exposure to Mg deficiency was used for analysis. Different dosages of Mg supply were categorized into low, medium and high Mg supply, where experimental design allowed for this clear differentiation between different Mg supplies. Study results measured on plants grown under sufficient or excessive Mg supply (compared to Mg deficient control) were assigned to high Mg supply. Studies that additionally included variation of nutrients other than Mg were included if there were no interactive effects. In the majority of studies, leaf Mg concentrations as well as net CO_2_ assimilation was measured in the fully expanded leaves. A few studies investigated differences between young and old leaves or did not specify the measured leaves at all (Table 1). These studies were included in the analysis by using a mean value. Computing an overall effect of leaf age was not possible without violating modeling assumptions due to the small sample size (limited number of studies) as well as the great diversity of these few studies.

Additional information on the studied plant species, cultivation method before onset of treatments, and plant age was extracted (Supplementary Material 1).

Studies where leaf Mg concentration was reported in combination with growth parameters or net CO2 assimilation were used to calculate critical leaf Mg concentration for growth. Leaf Mg concentration was related to growth parameters (dry weight, fresh weight, yield, height gain, relative growth rate) or net CO2assimilation to calculate critical leaf Mg concentrations for growth or photosynthesis, respectively. In cases where leaf Mg concentrations were missing, shoot Mg concentrations were used.

### Effect Size Metric and Data Analysis

For each individual comparison, the natural logarithm of the response ratio (lnR) was used to reflect relative changes in the respective parameter attributable to Mg nutrition:

(1) $$\ln\;\left(R\right)=\ln(\frac{T}{C})$$

Where T is the Mg treatment mean and C the control mean (no or clearly deficient Mg supply). If a Mg treatment increased the parameters in contrast to the control then the effect size lnR is positive. If a Mg treatment decreased the parameters, lnR is negative. The lnR and the variance of lnR were calculated using the escalc() function implemented in the metafor package (Viechtbauer, 2010) in R version 3.5.0 (R Core Team, 2018) with sample size as the weighting function (i.e., large sample size leads to greater weight within the analysis).

Random-effect models were fitted separately for effects on the different parameters. We used the function rma.mv() contained in the metafor package in R to perform different models with the moderators plant species, Mg supply (low, medium, high) and cultivation method (raising of plants under Mg supply or under Mg deficiency) while reference was set to random. Model outputs were back-transformed from lnR to relative changes (% of control) to compare treatments with the respective controls for specified moderators. Significance levels were set to 0.05. Model residuals were checked to verify homogeneity and normality.

To determine leaf Mg concentrations critical for growth or photosynthesis, the following equation was used:

(2) $$\begin{array}{r} y=a-\left(a-b\right)e^{-cx} \end{array}$$

y is net CO_2_ assimilation or the respective parameter for growth, while x is the leaf or shoot Mg concentration, respectively. The parameters a, b and c were estimated separately for each species using the nls() function in R.

## Results

### Effect of Magnesium Supply on Biomass

**Figure 1:**
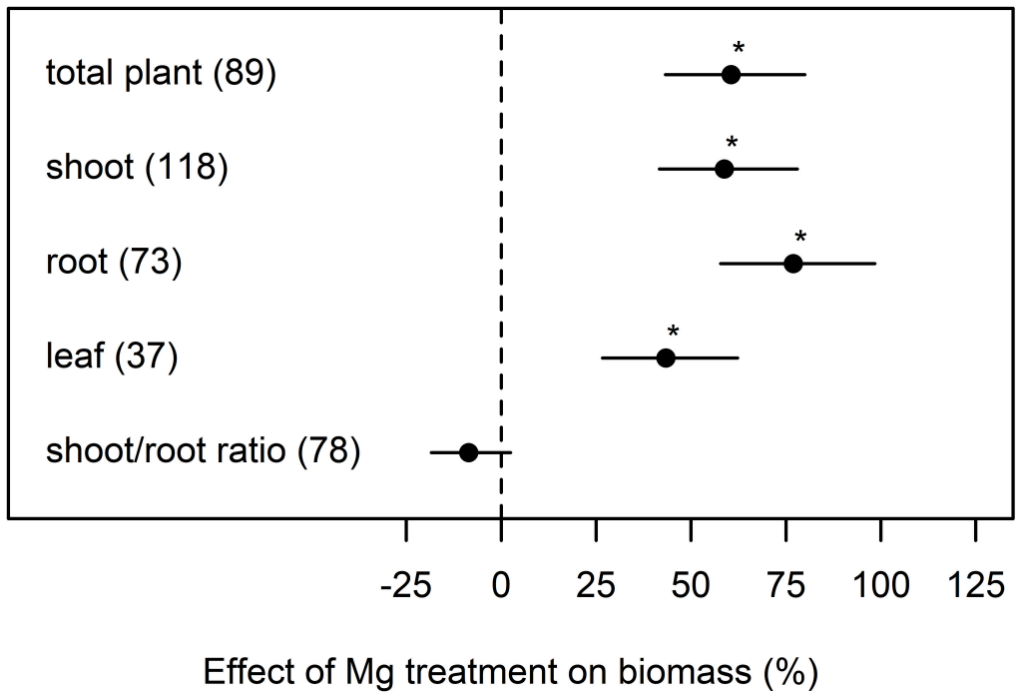
Effect of magnesium (Mg) supply on biomass of different plant parts compared to Mg deficient control (dashed line). Significant differences to the Mg deficient control are indicated with asterisks (*p* < 0.05). Numbers in brackets specify numbers of calculated effects.

Averaged over all studies that were included in our meta-analysis, Mg fertilization increased total plant biomass by 61% compared to Mg deficient control plants (Figure 1). The positive effect of Mg fertilization on the root biomass (77%) was greater than on shoot biomass (59%). On average, the shoot-root ratio was not significantly affected by Mg supply.

**Figure 2:**
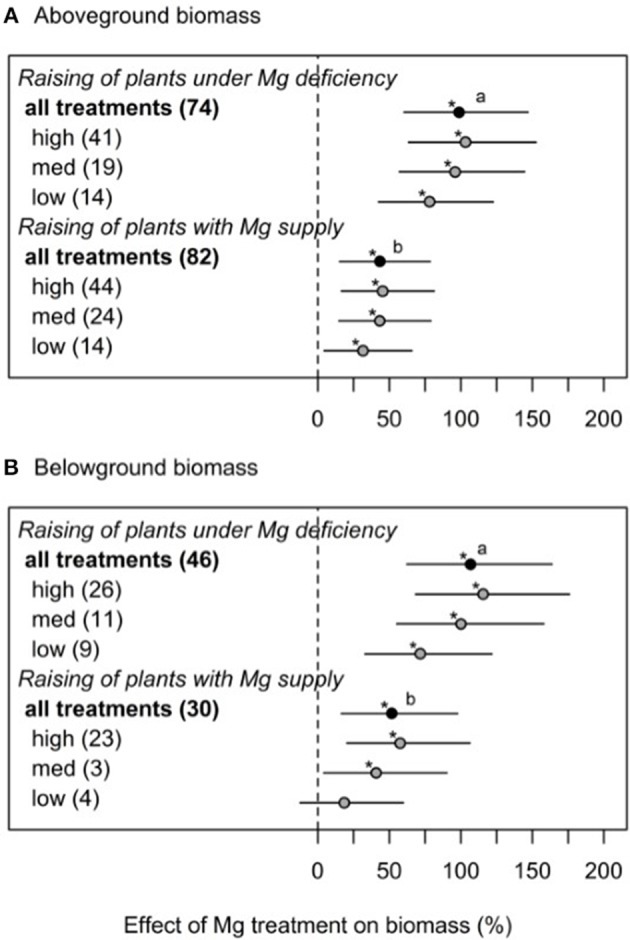
Effect of magnesium supply on above- **(A)** and belowground **(B)** biomass compared to Mg deficient control (dashed line). Trials are categorized into low, medium, high (=adequate and/or excessive) magnesium dosages and different cultivation methods (raising of plant with or without Mg deficiency prior to the onset of Mg treatments). Numbers in brackets specify numbers of calculated effects. Statistical differences between effects of moderators are indicated by different letters (*p* < 0.05). Significant differences to the Mg deficient control are indicated with asterisks (*p* < 0.05).

To further investigate the contradicting results between some studies—especially when regarding the Mg effect on shoot-root ratio—different experimental cultivation methods (cultivation of plants with and without Mg supply prior to the onset of deficiency treatments) as well as Mg dosages (low, medium, high) were categorized. If plants were initially raised under Mg deficiency, the effect of a subsequent Mg supply on shoot and root biomass was significantly greater (100% for shoot and 107% for root, respectively) than in plants with Mg supply before the onset of Mg deficiency treatments (43% for shoot and 52% for root biomass, respectively) (Figure 2).

**Figure 3:**
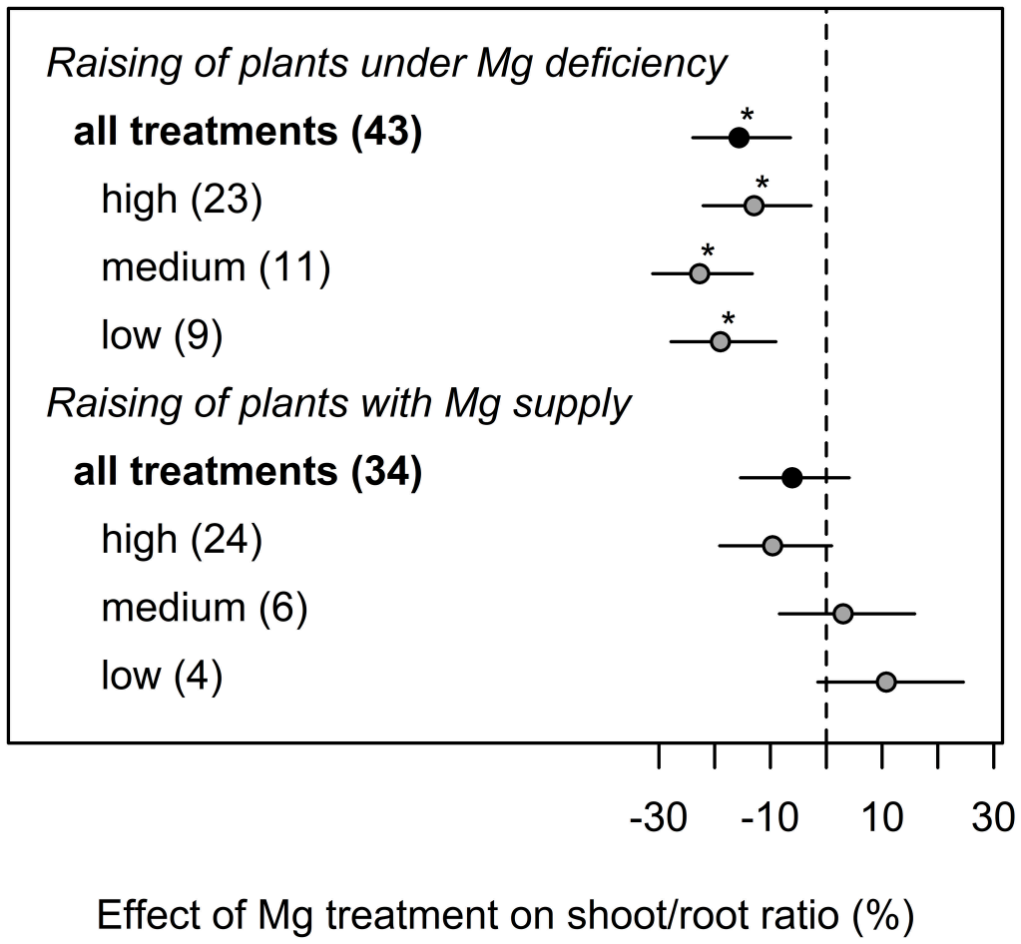
Effect of magnesium (Mg) supply on shoot-root ratio compared to the Mg deficient control (dashed line). Trials were categorized into low, medium, high (=adequate and/or excessive) Mg dosages and different cultivation methods (raising of plants with or without Mg deficiency before onset of deficiency treatments). Significant differences to the Mg deficient control are indicated with asterisks (*p* < 0.05). Numbers in brackets specify numbers of calculated effects. Data reported in Flores et al. (2015) were excluded from calculations underlying this figure because otherwise model assumptions would have been violated.

If plants were initially cultivated under Mg deficiency, shoot-root ratio was significantly decreased by 15% compared to Mg deficient plants (Figure 3). By contrast, no significant effect of Mg supply on shoot-root ratio compared to the control was observed when plants had been pre-cultured with adequate Mg supply.

### Effect of Mg Supply on Leaf Mg Concentration

**Figure 4:**
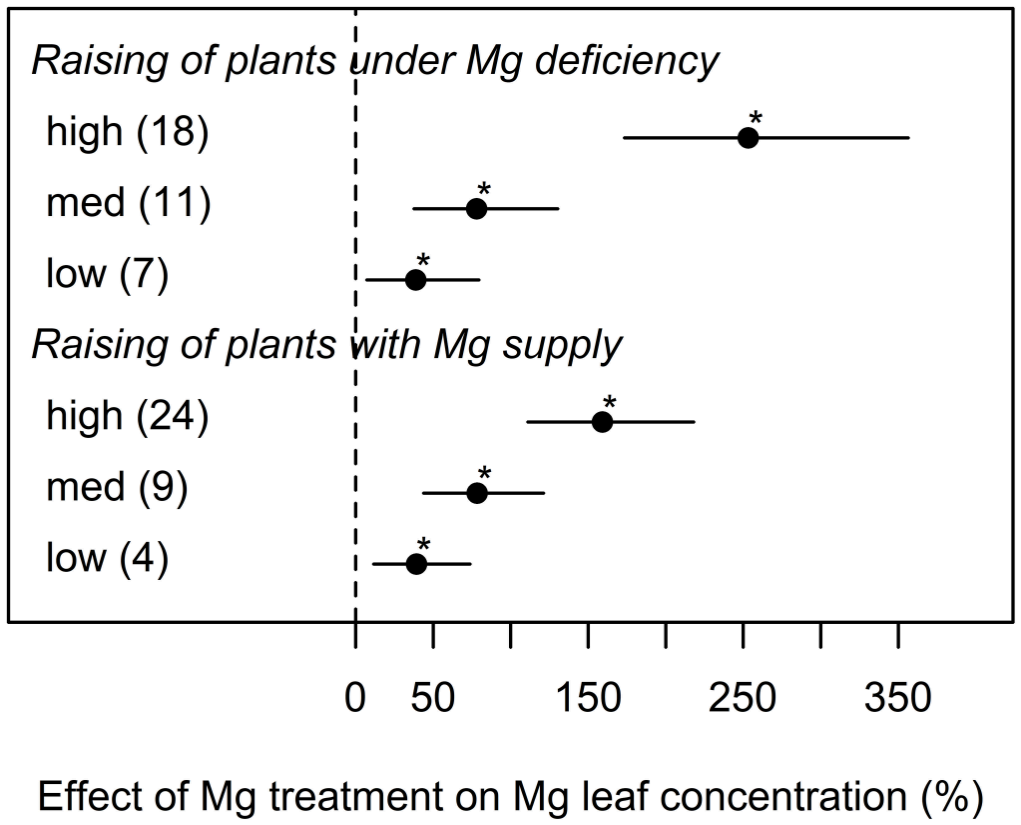
Effect of magnesium (Mg) supply on Mg leaf concentration compared to the Mg deficient control (dashed line). Trials were categorized into low, medium, high (=adequate and/or excessive) Mg dosages and different cultivation methods (raising of plant with or without Mg deficiency before onset of deficiency treatments). Significant differences to the Mg deficient control are indicated with asterisks (*p* < 0.05). Numbers in brackets specify numbers of calculated effects.

Magnesium concentrations in the leaf tissue were highly correlated with the amount of Mg that was supplied to the plant (Figure 4). However, unlike parameters of biomass formation, Mg leaf concentrations were not significantly affected by different experimental cultivation methods. In general, high Mg supply in trials where plants were raised under deficient Mg supply resulted in higher leaf Mg concentrations than in trials where plants were raised under sufficient amounts of Mg supply (Figure 4).

### Effect of Mg Supply on Net Assimilation and Photo-Oxidative Stress

Magnesium supply significantly enhanced net photosynthetic CO_2_ assimilation compared to Mg deficiency (Table 2). However, the effect of Mg treatment on net CO_2_ assimilation was not statistically different between medium and high levels of Mg supply.

**Table 2:** Effect of magnesium (Mg) supply on relative net assimilation (A_N_) compared to Mg deficient control (0).

**Mg treatment**	**Effect of Mg on A_**N**_ (% control)**	**CI**	**Number of effects**
Low	45b	6–99	8
Medium	127a	69–206	9
High	140a	79–222	29

*Trials were separated into low, medium, high (=adequate and/or excessive) Mg dosages. CI, Confidence interval. Different letters indicate significant differences between effects of moderators (p < 0.05)*.

**Figure 5:**
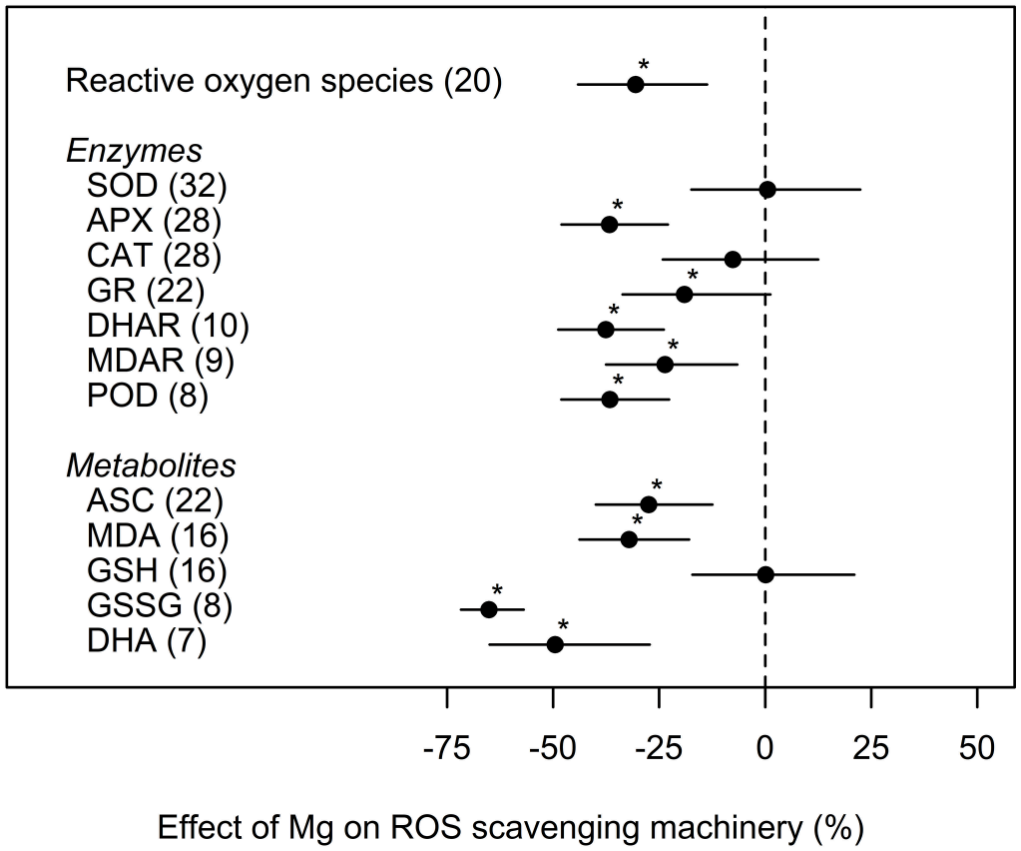
Effect of magnesium (Mg) fertilization on reactive oxygen species (ROS) and ROS scavenging enzymes and metabolites compared to Mg deficient control (dashed line). Significant differences to the Mg deficient control are indicated with asterisks (*p* < 0.05). Numbers in brackets specify numbers of calculated effects.

Concentrations of ROS were significantly reduced by 31% under medium and high Mg supply compared to deficient Mg supply. Similar effects were observed for most ROS scavenging enzymes and respective metabolites (Figure 5). The enzymes CAT and SOD and the metabolite GSH were not significantly influenced by Mg supply.

### Magnesium Leaf Concentrations Critical for Growth and Photosynthesis

**Figure 6:**
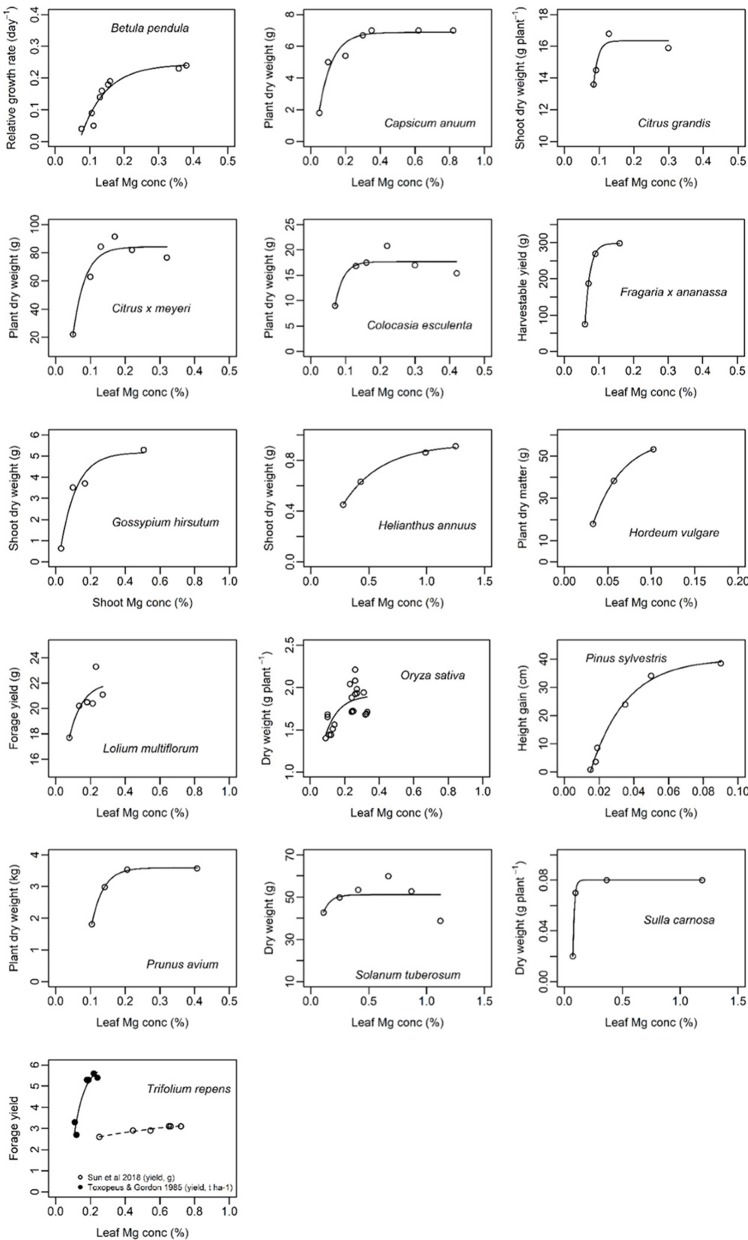
Biomass of different plant species in relation to leaf magnesium concentration (%). References of presented data are listed in Table 3. Curves of the function *y* = *a* − (*a* − *b*)*e*^−*cx*^ were fitted using nls() in R.

**Figure 7:**
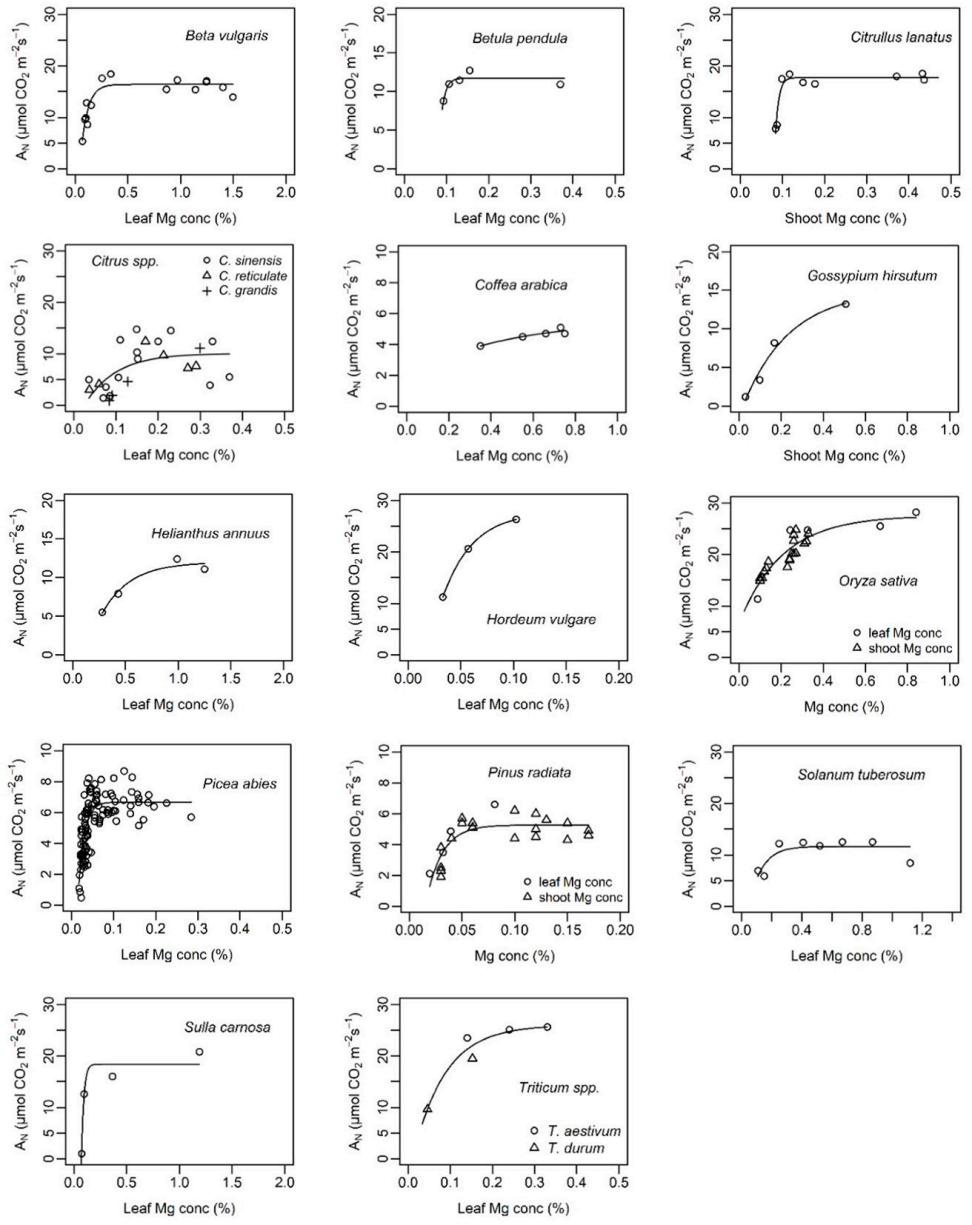
Net assimilation (A_N_) of different plant species in relation to leaf or shoot magnesium concentration (%). References of presented data are listed in Table 3. Curves of the function *y* = *a* − (*a* − *b*)*e*^−*cx*^ were fitted using nls() in R.

Generally, the relationship between leaf Mg concentration and biomass or net CO_2_ assimilation was highly species-specific and in some cases even study-specific (Figures 6, 7). Critical leaf Mg concentration for dry matter formation of woody plants ranged between 0.09 [*Citrus grandis* (L.) Osbeck] and 0.15% (*Malus domestica* L. Borkh.), in monocots between 0.07% (*Lolium perenne* L.) and 0.16-0.21% [*Sorghum bicolor* (L.) Moench] and in dicots between 0.10 (*Beta vulgaris* L., *Saccharum officinarum* L.) and 0.70% [*Perilla frutescens* (L.) Britton]. Critical leaf Mg concentrations of harvestable yield ranged between 0.08% for *Ribes nigrum* L. and 0.20% for *Trifolium repens* L. (Table 3). Critical leaf Mg concentration for net CO_2_ assimilation of woody species ranged between 0.05% (*Pinus* spp.) and 0.5% (*Coffea arabica* L.), in monocots between 0.02 (*Zea mays* L.) and 0.41% (*Oryza sativa* L.) and in dicots between 0.10 [*Citrullus lanatus* (Thunb.) Matsum. & Nakai] and 0.72% (*Helianthus annuus* L.). In general, critical Mg levels for legumes were higher than for most other species.

**Table 3:** Critical magnesium leaf concentrations for dry weight, harvestable yield, net CO_2_ assimilation (A_N_), and relative growth rate for different species.

**References**	**Species scientific name**	**Species common name**	**Critical Mg concentration (%) for**			
			**Dry weight**	**Harvestable yield**	**A_**N**_**	**Relative growth rate**
Fageria, 1976	*Arachis hypogaea* L.^‡^	Peanut	0.25–0.30^s^			
Terry and Ulrich, 1974	*Beta vulgaris* L.	Sugar beet	0.10		0.18	
Hermans et al., 2004	*Beta vulgaris* L.	Sugar beet	0.30^s^			
Ericcson and Kähr, 1995	*Betula pendula* Roth.	Birch			0.10^c^	0.24^c^
Riga and Anza, 2003	*Capsicum annuum* L.	Pepper	0.21			0.13
Huang et al., 2016	*Citrullus lanatus* (Thunb.) Matsum. and Nakai	Water melon			0.10^c^, ^s^	
Tang et al., 2012; Yang et al., 2012; Xiao et al., 2014; Peng et al., 2015; Jin et al., 2016; Li et al., 2017; Huang et al., 2018	*Citrus spp*.	Citrus			0.19^c^	
Yang et al., 2012	*Citrus grandis* (L.) Osbeck	Shaddock	0.09			
Trolove and Reid, 2012	*Citrus × meyeri*	Meyer's lemon	0.12			
Gonçalves et al., 2009	*Coffea arabica* L.	Coffee	0.11–0.12		0.50^c^	
Austin et al., 1994	*Colocasia esculenta* (L.) Schott	Taro	0.14^c^			
Hole and Scaife, 1993	*Daucus carota* L.	Carrot				0.56–0.75^p^
Bould, 1964	Fragaria × annanassa Duchesne	Strawberry		0.09^c^		
Melsted et al., 1969	*Glycine max* (L.) Merr.^‡^	Soybean	0.30			
Uzilday et al., 2017	*Gossypium hirsutum* L.	Cotton	0.23^c^, ^s^		0.48^c^, ^s^	
Lasa et al., 2000	*Helianthus annuus* L.	Sunflower	0.79^c^		0.72c	
Tränkner et al., 2016	*Hordeum vulgare* L.	Barley	0.10^c^		0.08^c^	
Sun et al., 2018	*Lolium multiflorum* Lam.	Italian ryegrass		0.13^c^		
Smith et al., 1985	*Lolium perenne* L.	Perennial ryegrass	0.07			
Bould and Parfitt, 1973	*Malus domestica* Borkh.	Apple	0.15			
Melsted et al., 1969	*Medicago sativa* L.^‡^	Alfalfa	0.40^s^			
Ding et al., 2006, 2008	*Oryza sativa* L.	Rice	0.17^c^		0.41^c^	
Fageria, 1976	*Oryza sativa* L.	Rice	0.12–0.17			
Choi and Park, 2008	*Perilla frutescens* (L.) Britton	Korean perilla	0.70^s^			
Sun and Payn, 1999; Laing et al., 2000; Sun et al., 2001	*Pinus radiata* D. Don	Monterey pine			0.05^c^	
Küppers et al., 1985	*Pinus sylvestris* L.	Scots pine				0.06^c^
Troyanos et al., 1997	*Prunus avium* L.	Wild cherry				0.16^c^
Bould, 1969	*Ribes nigrum* L.	Black currant		0.08		
Fageria, 1976	*Sacharum officinarum* L.	Sugar cane	0.10^s^			
Kasinath et al., 2014	*Solanum lycopersicum* L.	Tomato	0.39^p^			
Cao and Tibbitts, 1992(Koch et al., 2018)	*Solanum tuberosum* L.	Potato	0.14^c^		0.25^c^	
Grundon et al., 1987	*Sorghum bicolor* (L.) Moench	Sorghum	0.16–0.21			
Farhat et al., 2015, 2016b	*Sulla carnosa* Desf.^‡^	Sulla carnosa	0.10^c^		0.12^c^	
Debona et al., 2016; Yilmaz et al., 2017	*Triticum spp*.				0.18^c^	
Melsted et al., 1969	*Triticum aestivum* L.	Wheat	0.15^p^			
Toxopeus and Gordon, 1985	*Trifolium repens* L.^‡^	White clover		0.2^c^		
Maqbool et al., 2017	*Vaccinium angustifolium* Aiton	Blueberry	0.12–0.13^†^			
Hailes et al., 1997	*Zea mays* L.	Maize	0.15			
Walworth and Ceccotti, 1990	*Zea mays* L.	Maize	0.15^†^			

*Values were calculated from data reported in the listed references or cited, respectively. Critical is defined as 10% loss of maximal modeled value of the respective saturation curve (Figures 6, 7)*.

c *calculated*.

p *Mg plant concentration*.

s *Mg shoot concentration*.

† *Optimal Mg level*.

‡ *Legume*.

## Discussion

### Biomass Partitioning Under Mg Deficiency

Magnesium is involved in many biochemical and physiological processes in plants and critically contributes to photosynthesis and the subsequent transport of photo-assimilates (Cakmak and Marschner, 1992; Tränkner et al., 2018). In particular, the transport of sucrose from source leaves to sink organs such as roots is inhibited under Mg deficiency (Cakmak et al., 1994; Cakmak and Kirkby, 2008). This meta-analysis confirms the strong effect of Mg supply on shoot and whole plant biomass and an even more pronounced effect on root biomass (Figure 1). A reduced root biomass relative to shoots under Mg deficiency is considered as an early response to Mg deficiency because of the accumulation of carbohydrates in source leaves (Cakmak et al., 1994; Farhat et al., 2016a). However averaged over all 33 studies in our analysis, the shoot-root ratio was not affected by Mg supply, although an increased shoot-root ratio under Mg deficiency was often reported, e.g., in coffee (da Silva et al., 2014), bean (Cakmak et al., 1994), wheat and maize (Mengutay et al., 2013). In contrast, the shoot-root ratio did not significantly change in other studies in response to Mg nutrition e.g., in *Arabidopsis* (Hermans and Verbruggen, 2005), sugar beet (Hermans et al., 2005), maize (Kumar Tewari et al., 2004) and barley (Tränkner et al., 2016), and in Mg deficient rice, a decrease in shoot-root ratio was described (Ding et al., 2006).

To explore the reasons for contradictory effects of Mg treatments on shoot-root ratio, we explored the database for studies where plants were raised under Mg deficiency compared to those raised with adequate Mg supply before the onset of Mg treatments. As suggested by Tränkner et al. (2016) and Verbruggen and Hermans (2013), when Mg is available in very early growth stages, the shoot-root ratio was not affected even when plants ran into Mg deficiency later. This is often the case in scientific studies conducted in hydroponic culture, where plants are initially grown under adequate Mg supply before Mg concentration is reduced. However, the present study shows that Mg concentrations in the leaf tissue of Mg deficient plants are similarly affected when plants were raised with Mg supply compared to plants raised under deficient Mg supply. Thus, we conclude from these data, that restricted growth assigned to Mg deficiency is most severe if deficiency occurs during very early developmental stages. In this context, Kobayashi et al. (2013) investigated differential time courses of symptom appearance in leaves of young Mg deficient rice plants followed by Mg resupply. They concluded that there is a time frame of recoverability from Mg deficiency, indicating the existence of a period of extreme sensitivity to low Mg concentrations depending on the leaf growing stage. Magnesium can be stored in great amounts within vacuoles. In Norway spruce, vacuolar Mg concentrations of up to 120 mM in endodermis cells and up to 17 mM Mg^2+^ in mesophyll cells were reported (Hawkesford et al., 2012). High vacuolar Mg concentrations serve as a pool for maintaining Mg homeostasis in other cells during growth (Stelzer et al., 1990). Furthermore, Mg is very mobile within the plant and older plant parts may serve as a Mg pool for younger, growing plant parts with a high demand for Mg. Plants that were initially grown under adequate Mg supply most likely have stored enough Mg in these Mg pools to withstand upcoming deficiency situations to a certain degree—resulting in a lesser reduction of growth even if availability of Mg is suddenly limited.

Interestingly, Andrews et al. (1999) found—under similar experimental conditions—an increased shoot-root ratio of Mg deficient pea and bean, while the shoot-root ratio of wheat decreased under Mg deficiency. In their regression model, leaf soluble protein concentration was positively correlated to shoot-root ratio and could explain most of the variation in the shoot-root ratio. Leaf soluble protein reflect the availability of nitrogen (N) substrate for growth. Moreover, Mg is considered important for the uptake of nitrogen and the nitrogen use efficiency (Grzebisz, 2013). Results of Andrews et al. (1999) indicate that the impaired root growth relative to shoots under Mg deficiency (or other deficiencies) may be a result of N limitation rather than an inhibited transport of carbohydrates. Data provided by Lasa et al. (2000) also support this hypothesis: both, leaf soluble protein content and the shoot-root ratio decreased with increasing Mg supply in ammonium fed sunflower, while in nitrate fed sunflower, leaf soluble protein as well as shoot-root ratio did not change under different levels of Mg supply (Lasa et al., 2000).

### Importance of Mg for CO_2_ Assimilation and Photo-Oxidative Defense

The importance of an adequate Mg supply for photosynthetic CO_2_ assimilation was confirmed by our study (Table 2). Mg directly affects Rubisco activity and activation by binding to the carbamylated Rubisco side chain and to the catalytic chaperone Rubisco activase (Hazra et al., 2015), hence less Mg supply decreases net CO_2_ assimilation rates. Numerous reports emphasize the positive effect of Mg supply on net CO_2_ assimilation of various species and under various growing conditions, for instance, in hydroponic systems (Laing et al., 2000; Ding et al., 2006; Tränkner et al., 2016; Samborska et al., 2018), soil or sand filled pots (Yang et al., 2012; Canizella et al., 2015; Peng et al., 2015) or in the field (Tang et al., 2012). Only in cases where very early Mg deficiency symptoms were studied was there no effect of Mg on net CO_2_ assimilation or photosynthetic capacity (Chen C. T. et al., 2018; Koch et al., 2018). Net CO_2_ assimilation was found to increase with increasing leaf Mg concentration showing typical curvilinear species-specific response curves with maximum assimilation above a certain threshold of Mg concentrations (Figure 7).

Restricted assimilation under Mg shortage is known to increase oxidative stress because electrons and excitation energy not used in photosynthesis induce excessive production of ROS in cell compartments. Accordingly, we found that levels of ROS increased by 31% under Mg deficiency (Figure 5). Interestingly, recent findings by Kobayashi et al. (2018) showed an increased oxidative stress in Mg deficient rice plants before any decrease in net CO_2_ assimilation was detected, i.e., independently of the carbohydrate metabolism. They suppose that decreased Mg concentrations may trigger excess iron stress at the cellular level by disrupting mechanisms for immobilizing toxic iron ions into the vacuole of expanding young leaf cells.

However, to protect cells against photo-oxidation by ROS, plants have developed a complex antioxidant system. Thus, an increased activity of the anti-oxidative system, including increased activity of ROS-detoxifying enzymes, are generally expected under Mg deficiency. This was confirmed by our analyses concerning the enzymes APX, GR, DHAR, MDAR, and POD. Their activities were decreased in accordance with a mitigation of ROS levels in case of an adequate Mg supply (Figure 5). Nonetheless, highly contrasting results on the activity of the important antioxidants CAT and SOD are reported. Hence, our meta-analysis could not identify any trend toward a significant increase or decrease of their activities due to Mg deficiency. Authors provide different explanations for contradictory results in enzyme activities during ROS scavenging. For instance, Ding et al. (2008) speculates that the degree of nutrient deficiency might be responsible for the activation or not of the oxidative stress defense system. Plants suffering severe nutrient stress might lose the proper response to the stress.

Tang et al. (2012) report lower CAT activity (combined with lower Rubisco activity) in Mg deficient leaves. The authors supposed that their findings may reflect a lower rate of photorespiration in Mg-deficient leaves, as CAT is primarily localized in the peroxisome, where it is involved in removing the bulk H_2_O_2_ generated by photorespiration. Similarly, several authors report a decreased activity of CAT in Mg-deficient leaves whereas activities of other antioxidant enzyme were increased (Kumar Tewari et al., 2006; Yang et al., 2012; Mengutay et al., 2013). They relate the Mg-deficiency-induced decrease in CAT activity to the fact that CAT is sensitive to photo-inactivation *in vivo* and *in vitro*. Hence, photo-inactivation of CAT may be caused by oxidative damage that is initiated in the chloroplast via direct absorption of light by the heme moieties of the enzyme itself (Shang and Feierabend, 1999). Additionally, CAT is known to be active only at relatively high H_2_O_2_ concentrations. Lower H_2_O_2_ concentrations are eliminated by APX or other peroxidases with the aid of various reductants such as ascorbate and glutathione (Gechev et al., 2006).

Unlike for CAT, detailed explanations for the sometimes reduced activity of SOD under Mg deficiency as reported by several authors (Polle et al., 1994; Ze et al., 2009b; Zhang et al., 2015; Rehman et al., 2018) are so far missing. SOD is the first line of defense involved in the ROS scavenging process as it converts superoxide radicals to H_2_O_2_. The other antioxidant enzymes then detoxify H_2_O_2_. For instance, Polle et al. (1994) found a decreased SOD activity under Mg deficiency together with chlorosis in spruce needles while other antioxidant enzyme activities were increased and relate their finding to a failure of SOD under Mg deficiency rather than insufficient detoxification of H_2_O_2_. On the other hand, Ze et al. (2009b) found a general inhibition of the anti-oxidative defense system under Mg deficiency together with an increased ROS production. Thus, a general inhibition of the anti-oxidative defense system under certain degrees of Mg deficiency is supposed.

Nonetheless, the ROS–antioxidant balance within different compartments of photosynthetically active cells is a highly dynamic system (temporal as well as spatial) and might therefore not be adequately reflected by static measurements. For instance, studies that investigated time courses of ROS accumulation and enzyme activities during the onset of Mg deficiency found different antioxidant enzyme activities as well as ascorbate, glutathione or ROS concentrations depending on the time after the onset of Mg deficiency (Anza et al., 2005; Alsharafa, 2017; da Silva et al., 2017). Hence, contradictory results might also be explained by different experimental setups and further dependencies e.g., on the sampling date or day time of sampling or interrelations to other metabolic cycles such as nitrogen assimilation and photorespiration are likely. Although our meta-analysis is the first that comprised overall effects of Mg deficiency on ROS and the ROS scavenging machinery, the reasons for contradictory results especially concerning the important antioxidant SOD remain unclear and further research is needed.

### Critical Mg Concentration for Net CO_2_ Assimilation and Biomass

Leaf nutrient concentrations are an important diagnostic tool for site-specific and efficient nutrient management. In practice, critical thresholds of nutrient concentrations specify the level of nutrition below which significant yield losses are to be expected. However, determining critical Mg concentrations is challenging because Mg concentrations in a specific species may vary due to the growing condition, growth stage, genotype/variety, presence/absence of antagonistic nutrients (mainly Ca and K) or the age of the analyzed tissue (Gransee and Führs, 2013). Differences in leaf Mg concentrations under Mg deficiency due to the leaf age were observed in e.g., bean (Neuhaus et al., 2014), barley (Tränkner et al., 2016), mulberry (Kumar Tewari et al., 2006) and Norway spruce (Mehne-Jakobs, 1996). In contrast, Austin et al. (1994) found no differences between old and young leaves of taro. Furthermore, dose-response curves are needed in order to detect critical Mg concentrations for CO_2_ assimilation or yield. The question arises if these dose-response curves and therefore, the respective critical Mg concentrations may depend on the above listed factors, i.e., on the growing condition, the age of the sampled leaf, the growing stage etc. In this context, Mehne-Jakobs (1996) plotted net CO_2_ assimilation of Norway spruce vs. leaf Mg concentration and found a physiological threshold for net CO_2_ assimilation of 0.4 mg Mg g^−1^ dry weight independently of the leaf age (1 year old and current-year needles). Furthermore, in our study, we combined data of seven studies investigating different species of *Citrus* (studies differed in experimental conditions and plant growing stages) and found that the data fit into the same model that could be used to determine the critical Mg threshold for net CO_2_ assimilation of *Citrus* (Figure 7). This indicates, that the leaf age, growing condition or plant growing stage might be of minor relevance for the critical Mg thresholds of net CO_2_ assimilation as the photosynthetic performance of a leaf primary depends on Mg tissue concentration (assuming other stresses or nutrient deficiencies can be excluded).

Although numerous studies addressed the physiologic and phenotypic response of plants to Mg deficiency (e.g., Terry and Ulrich, 1974; Cakmak et al., 1994; Hermans et al., 2005; Yang et al., 2012; Farhat et al., 2015; Huang et al., 2016; Li et al., 2017), critical Mg levels reported for growth or photosynthesis are either study-specific or not available at all for a lot of species. In the present study, we combined all scientifically published data that relate Mg concentrations to growth parameters. We found a critical leaf Mg range for dry weight between 0.1 and 0.2% for numerous crop species such as wheat, potato, rice, maize, sorghum and barley (Table 3). Leaf Mg concentrations critical for dry matter formation of species with a generally higher Mg demand range between 0.2 and 0.3% (e.g., cotton, soybean or peanut), and even above 0.35% for alfalfa, sunflower and tomato. The reason for variations in Mg demand by specific species is unclear. Liu et al. (2014) described differences in Mg concentrations between different plant functional types in a karst ecosystem in China. They found a significantly higher Mg concentration in green leaves of deciduous shrubs, ferns or forbs than in evergreen trees or grasses. In the present meta-analysis, we attempted to extend species-specific critical Mg thresholds to broader groups of species: therefore, data were grouped into e.g., legumes vs. non-legumes, monocots vs. dicots, or annual vs. perennial. However, explanatory power of the respective models was low because of the limited number of species per group (data not shown). Nonetheless, we found that legume species have higher critical leaf Mg concentrations and monocots lower critical leaf concentrations than most other species, respectively.

Instead of critical threshold concentrations, optimal Mg ranges for crop growth are sometimes reported. Walworth and Muniz (1993) reviewed the effect of leaf Mg concentrations on potato growth and report optimal levels between 0.26 and 1.25%, and low levels between 0.15 and 0.25%—low meaning that the plant will be responsive to fertilization. This finding is in accordance with the critical Mg level for potato of 0.14% that was calculated in the present study. By contrast with the findings of Walworth and Muniz (1993), we observed a decline in biomass as well as net assimilation of potato for high Mg concentrations above 1%. Similarly, Gunes et al. (1998) who aimed at identifying critical plant nutrient concentrations for growth of tomato plants, found decreasing dry matter with increasing Mg concentration. However, in their study, Mg concentrations ranged between 0.71 and 1.62%, which is rather in a super-optimal range regarding reports from Kasinath et al. (2014) who found a critical Mg level for growth of tomato at 0.39%. Hence, Mg concentrations of 1.62% in tomato might induce deficiency in nutrients which are antagonistic to Mg, as high Mg uptake can significantly decrease the uptake of K and Ca (Fageria, 2001) and thereby limiting dry matter production.

Interestingly, calculated and cited critical levels for net CO_2_ assimilation were mostly higher than for biomass or harvestable yield (Table 3) as also described by Terry and Ulrich (1974). On the one hand, this implies that physiologically important processes for plant growth can be already negatively affected—although Mg supply is not limiting biomass or yield. On the other hand, it implies that maximal net CO_2_ assimilation is not a prerequisite for maximal dry matter or yield. However, critical Mg levels where mostly investigated under laboratory or greenhouse conditions, where complex interactions between various biotic and abiotic stress situations are excluded. Especially under high light intensities together with high rates of vegetative growth, plant requirements for Mg are high. In the field, where multiple stress factors are likely to occur, even slight Mg deficiency may affect plant susceptibility to environmental stresses and therefore biomass formation. Hence, critical Mg levels for CO_2_ assimilation rather than for crop growth parameters should be used to define the plant Mg status, since it allows early recognition of Mg stress before biomass formation is affected.

Our study confirmed that plants—independently of the species—react to adequate Mg supply with lower levels of oxidative stress and higher rates of net CO_2_ assimilation. Biomass partitioning between shoot and roots was affected by the cultivation technique: the shoot-root ratio was significantly increased only if plants were initially grown without Mg supply. The effect of Mg supply on root biomass was similar than that on shoot biomass in case plants were initially grown with Mg before exposing them to Mg deficiency.

In conclusion, our evaluation can be used to optimize fertilization strategies as it shows the specific requirement of Mg for various crop and tree species for maintaining physiologically important processes such as net CO_2_ assimilation, which is important for optimal plant growth and productivity.

## Author Contributions

MT conceived the presented idea. MH-J performed the literature search, data extraction, statistical analyses, and data presentation. MT supervised the findings of this work. All authors contributed to the final manuscript.

### Conflict of Interest Statement

The authors declare that the research was conducted in the absence of any commercial or financial relationships that could be construed as a potential conflict of interest.
