# Supplementary material for: Critical Leaf Magnesium Thresholds and the Impact of Magnesium on Plant Growth and Photo-Oxidative Defense: A Systematic Review and Meta-Analysis From 70 Years of Research
Source: Front Plant Sci. 2019 Jun 18;10:766. doi: 10.3389/fpls.2019.00766 (PMC6592071; doi:10.3389/fpls.2019.00766)
Supplement: Supplementary file 2 [file Data_Sheet_1.pdf]

Supplementary material 2: References included in the meta-analysis

Alsharafa, K. Y. (2017). Mineral deficiencies influence on tomato leaves: Pigments, hydrogen peroxide and total phenolic compounds contents. *Plant OMICS*, 10(2), 78–87. https://doi.org/10.21475/poj.10.02.17.pne386

Anza, M., Riga, P., & Garbisu, C. (2005). Time course of antioxidant responses of *Capsicum annuum* subjected to a progressive magnesium deficiency. *Annals of Applied Biology*, 146(1), 123–134. https://doi.org/10.1111/j.1744-7348.2005.04023.x

Austin, M. T., Constantinides, M., Miyasaka, S. C. (1994). Effect of magnesium on early taro growth. *Communications in Soil Science and Plant Analysis*, 25(11-12), 2159-2169.

Blasco, B., Graham, N. S., Broadley, M. R. (2015). Antioxidant response and carboxylate metabolism in *Brassica rapa* exposed to different external Zn, Ca, and Mg supply. *Journal of Plant Physiology*, 176, 16–24.

Cakmak, I., Marschner, H. (1992). Magnesium deficiency and high light intensity enhance activities of superoxide dismutase, ascorbate peroxidase, and glutathione reductase in bean leaves. *Plant Physiology*, 98, 1222–1227.

Cakmak, I., Hengeler, C., Marschner, H. (1994). Partitioning of shoot and root dry matter and carbohydrates in bean plants suffering from phosphorus, potassium and magnesium deficiency. *Journal of Experimental Botany*, 45, 1245–1250.

Chao, Y. Y., Chou, T. S., & Kao, C. H. (2012). Involvement of abscisic acid and hydrogen peroxide in regulating the activities of antioxidant enzymes in leaves of rice seedlings under magnesium deficiency. *Plant Growth Regulation*, 66(1), 1–8. https://doi.org/10.1007/s10725-011-9623-9

Chen, J., Li, Y., Wen, S., Rosanoff, A., Yang, G., & Sun, X. (2017). Magnesium fertilizer-induced increase of symbiotic microorganisms improves forage growth and quality. *Journal of Agricultural and Food Chemistry*, 65(16), 3253–3258. https://doi.org/10.1021/acs.jafc.6b05764

Chou, T. S., Chao, Y. Y., Huang, W. D., Hong, C. Y., & Kao, C. H. (2011). Effect of magnesium deficiency on antioxidant status and cadmium toxicity in rice seedlings. *Journal of Plant Physiology*, 168(10), 1021–1030. https://doi.org/10.1016/j.jplph.2010.12.004

da Silva, D. M., Brandão, I. R., Alves, J. D., de Santos, M. O., de Souza, K. R. D. & de Silveira, H. R. O. (2014). Physiological and biochemical impacts of magnesium-deficiency in two cultivars of coffee. *Plant and Soil*, 382, 133–150.

da Silva, D. M., de Souza, K. R. D., Vilas Boas, L. V., Alves, Y. S., & Alves, J. D. (2017). The effect of magnesium nutrition on the antioxidant response of coffee seedlings under heat stress. *Scientia Horticulturae*, 224, 115–125.

de Almeida, T. B. F., Flores, R. A., de Almeida, H. J., de Mello Prado, R., Maranhão, D. D. C., & Politi, L. S. (2017). Development and nutrition of soybeans with macronutrients deficiencies. *Communications in Soil Science and Plant Analysis*, 48(13), 1616–1625. https://doi.org/10.1080/00103624.2017.1374402

Dias, K. G. de L., Guimarães, P. T. G., Neto, A. E. F., Silveira, H. R. O. de, & Lacerda, J. J. de J. (2017). Effect of magnesium on gas exchange and photosynthetic efficiency of coffee plants grown under different light levels. *Agriculture*, 7(10), 85. https://doi.org/10.3390/agriculture7100085

Ding, Y. C., Chang, C. R., Luo, W., Wu, Y. S., Ren, X. L., Wang, P. & Xu, G. H. (2008). High potassium aggravates the oxidative stress induced by magnesium deficiency in rice leaves. *Pedosphere*, 18, 316–327.

Farhat, N., Elkhouni, A., Zorrig, W., Smaoui, A., Abdelly, C., & Rabhi, M. (2016a). Effects of magnesium deficiency on photosynthesis and carbohydrate partitioning. *Acta Physiologiae Plantarum, 38: 145.* https://doi.org/10.1007/s11738-016-2165-z

Farhat, N., Ivanov, A. G., Krol, M., Rabhi, M., Smaoui, A., Abdelly, C., & Hüner, N. P. A. (2015). Preferential damaging effects of limited magnesium bioavailability on photosystem I in *Sulla carnosa* plants. *Planta*, 241(5), 1189-1206. https://doi.org/10.1007/s00425-015-2248-x

Farhat, N., Smaoui, A., Maurousset, L., Porcheron, B., Lemoine, R., Abdelly, C., & Rabhi, M. (2016b). *Sulla carnosa* modulates root invertase activity in response to the inhibition of long-distance sucrose transport under magnesium deficiency. *Plant Biology*, 18(6), 1031–1037. https://doi.org/10.1111/plb.12489

Farzadfar, S., Zarinkamar, F., & Hojati, M. (2017). Magnesium and manganese affect photosynthesis, essential oil composition and phenolic compounds of *Tanacetum parthenium*. *Plant Physiology and Biochemistry*, 112, 207–217. https://doi.org/10.1016/j.plaphy.2017.01.002

Fischer, E. S., Lohaus, G., Heineke, D., & Weldt, H. W. (1998). Magnesium deficiency results in acculumation of carbohydrates and amino acids in source and sink leaves of spinach. *Physiologia Plantarum*, 102, 16–20.

Flores, R. A., Borges, B. M. M. N., Almeida, H. J. & Prado, R. D. M. (2015). Growth and nutritional disorders of eggplant cultivated in nutrients solutions with suppressed macronutrients. *Journal of Plant Nutrition*, 38(7), 1097-1109.

Hariadi, Y., & Shabala, S. (2004b). Screening broad beans (*Vicia faba*) for magnesium deficiency. II. Photosynthetic performance and leaf bioelectrical responses. *Functional Plant Biology*, 31(5), 539–549. https://doi.org/10.1071/FP03202

Hermans, C., Johnson, G. N., Strasser, R. J. & Verbruggen, N. (2004). Physiological characterisation of magnesium deficiency in sugar beet: acclimation to low magnesium differentially affects photosystems I and II. *Planta*, 220, 344–355.

Hermans, C. & Verbruggen, N. (2005). Physiological characterization of Mg deficiency in *Arabidopsis thaliana*. *Journal of Experimental Botany*, 56, 2153-2161.

Huang, Y., Jiao, Y., Nawaz, M. A., Chen, C., Liu, L., Lu, Z., Kong, Q., Cheng, F. & Bie, Z. (2016). Improving magnesium uptake, photosynthesis and antioxidant enzyme activities of watermelon by grafting onto pumpkin rootstock under low magnesium. *Plant and Soil*, 409, 229–246.

Jezek, M., Geilfus, C. M., Bayer, A., Mühling, K. H. & Struik, P. C. (2015). Photosynthetic capacity, nutrient status, and growth of maize (*Zea mays* L.) upon $MgSO_4$ leaf-application. *Frontiers in Plant Science*, 5, 1–10.

Jin, X.-L., Ma, C.-L., Yang, L.-T. & Chen, L.-S. (2016). Alterations of physiology and gene expression due to long-term magnesium-deficiency differ between leaves and roots of *Citrus reticulata*. *Journal of Plant Physiology*, 198, 103-115. http://dx.doi.org/10.1016/j.jplph.2016.04.011

Jung, I., & Winter, K. (1992). Mineral nutrient deficiency increases the sensitivity of photosynthesis to sulphur dioxide in needles of a coniferous tree, *Abies nordmanniana*. *Oecologia*, 90(1), 70–73. https://doi.org/10.1007/BF00317810

Koch, M., Busse, M., Naumann, M., Jákli, B., Smit, I., Cakmak, I., Hermans, C. & Pawelzik, E. (2018). Differential effects of varied potassium and magnesium nutrition on production and partitioning of photoassimilates in potato plants. *Physiologia Plantarum*. https://doi.org/10.1111/ppl.12846

Kumar Tewari, R., Kumar, P., & Nand Sharma, P. (2006). Magnesium deficiency induced oxidative stress and antioxidant responses in mulberry plants. *Scientia Horticulturae,* 108, 7-14. https://doi.org/10.1016/j.scienta.2005.12.006

Kumar Tewari, R., Kumar, P., Tewari, N., Srivastava, S., & Sharma, P. N. (2004). Macronutrient deficiencies and differential antioxidant responses - Influence on the activity and expression of superoxide dismutase in maize. *Plant Science*, 166(3), 687–694. https://doi.org/10.1016/j.plantsci.2003.11.004

Küppers, M., Zech, W., Schulze, E.-D. & Beck, E. (1985). CO_2-Assimliation, Transpiration und Wachstum von *Pinus silvestris* L. bei unterschiedlicher Magnesiumversorgung. *European Journal of Forest Research*, 104, 23-36.

Kwano, B. H., Moreira, A., Moraes, L. A. C., & Nogueira, M. A. (2017). Magnesium-manganese interaction in soybean cultivars with different nutritional requirements. *Journal of Plant Nutrition*, 40(3), 372–381. https://doi.org/10.1080/01904167.2016.1240198

Lasa, B., Frechilla, S., Aleu, M., González-Moro, B., Lamsfus, C., & Aparicio-Tejo, P. M. (2000). Effects of low and high levels of magnesium on the response of sunflower plants grown with ammonium and nitrate. *Plant and Soil*, 225(1–2), 167–174. https://doi.org/10.1023/A:1026568329860

Li, C. P., Qi, Y. P., Zhang, J., Yang, L. T., Wang, D. H., Ye, X., … Chen, L. S. (2017). Magnesium-deficiency-induced alterations of gas exchange, major metabolites and key enzymes differ among roots, and lower and upper leaves of *Citrus sinensis* seedlings. *Tree Physiology*, 37(11), 1564–1581. https://doi.org/10.1093/treephys/tpx067

Mehne-Jakobs, B. (1995). Seasonal development of the photosynthetic performance of Norway spruce (*Picea abies* [L.] Karst.) under magnesium deficiency. *Plant and Soil*, 168–169(1), 255– 261. https://doi.org/10.1007/BF00029336

Mengutay, M., Ceylan, Y., Kutman, U. B., & Cakmak, I. (2013). Adequate magnesium nutrition mitigates adverse effects of heat stress on maize and wheat. *Plant and Soil*, 368(1–2), 57–72. https://doi.org/10.1007/s11104-013-1761-6

Moreira, W. R., Bispo, W. M. da S., Rios, J. A., Debona, D., Nascimento, C. W. A., & Rodrigues, F. Á. (2015). Magnesium-induced alterations in the photosynthetic performance and resistance of rice plants infected with *Bipolaris oryzae*. *Scientia Agricola*, 72(4), 328–333. https://doi.org/10.1590/0103-9016-2014-0312

Neuhaus, C., Geilfus, C. M., & Mühling, K. H. (2014). Increasing root and leaf growth and yield in Mg-deficient faba beans (*Vicia faba*) by $MgSO_4$ foliar fertilization. *Journal of Plant Nutrition and Soil Science, 177(5), 741-747*. https://doi.org/10.1002/jpln.201300127

Neuhaus, C., Geilfus, C. M., Zörb, C., & Mühling, K. H. (2013). Transcript expression of Mg-chelatase and H+-ATPase isogenes in *Vicia faba* leaves as influenced by root and foliar magnesium supply. *Plant and Soil*, 368(1–2), 41–50. https://doi.org/10.1007/s11104-013-1711-3

Niu, Y. F., Jin, G. L., & Zhang, Y. S. (2014). Root development under control of magnesium availability. *Plant Signaling and Behavior*, 9:e29720, 1–5. https://doi.org/10.4161/psb.29720

Orlovius, K. & McHoul, J. (2015): Effect of two magnesium fertilizers on leaf magnesium concentration, yield, and quality of potato and sugar beet. *Journal of Plant Nutrition*, 38. https://doi.org/10.1080/01904167.2014.958167

Pal, P. K., & Mahajan, M. (2017). Pruning system and foliar application of $MgSO_4$ alter yield and secondary metabolite profile of *Rosa damascena* under rainfed acidic conditions. *Frontiers in Plant Science,* 8:507. https://doi.org/10.3389/fpls.2017.00507

Peng, H. Y., Qi, Y. P., Lee, J., Yang, L. T., Guo, P., Jiang, H. X., & Chen, L. S. (2015). Proteomic analysis of *Citrus sinensis* roots and leaves in response to long-term magnesium-deficiency. *BMC Genomics*, 16(1), 1–24. https://doi.org/10.1186/s12864-015-1462-z

Pobereżny, J., Wszelaczyńska, E., & Keutgen, A. J. (2012). Yield and chemical content of carrot storage roots depending on foliar fertilization with magnesium and duration of storage*. Journal of Elementology*, 3, 479–494. https://doi.org/10.5601/jelem.2012.17.3.10

Polle, A., Otter, T., & Mehne Jakobs, B. (1994). Effect of magnesium deficiency on anti-oxydative systems in needles of Norway spruce (*Picea abies* (L.) Karst.) grown with different ratios of nitrate and ammonium as nitrogen sources. *New Phytologist*, 128, 621–628.

Radkowski, A., Radkowska, I., Rapacz, M., & Wolski, K. (2017). Effect of foliar fertilization with magnesium sulfate and supplemental L-ascorbic acid on dry matter yield and chemical composition of cv. Egida timothy grass. *Journal of Elementology*, 22(2), 545–558. https://doi.org/10.5601/jelem.2016.21.3.1167

Rehman, H. ur, Alharby, H. F., Alzahrani, Y., & Rady, M. M. (2018). Magnesium and organic biostimulant integrative application induces physiological and biochemical changes in sunflower plants and its harvested progeny on sandy soil. *Plant Physiology and Biochemistry*, 126, 97–105. https://doi.org/10.1016/j.plaphy.2018.02.031

Riga, P., & Anza, M. (2003). Effect of magnesium deficiency on pepper growth parameters: Implications for determination of magnesium-critical value. *Journal of Plant Nutrition*, 26(8), 1581–1593. https://doi.org/10.1081/PLN-120022367

Rivelli, A. R., de Maria, S., Pizza, S., & Gherbin, P. (2010). Growth and physiological response of hydroponically-grown sunflower as affected by salinity and magnesium levels. *Journal of Plant Nutrition*, 33(9), 1307–1323. https://doi.org/10.1080/01904167.2010.484092

Ruan, J., & Gerendás, J. (2015). Absorption of foliar-applied urea-^{15}N and the impact of low nitrogen, potassium, magnesium and sulfur nutritional status in tea (*Camellia sinensis* L.) plants. *Soil Science and Plant Nutrition*, 61(4), 653–663. https://doi.org/10.1080/00380768.2015.1027134

Ruan, J., Ma, L., & Yang, Y. (2012). Magnesium nutrition on accumulation and transport of amino acids in tea plants. *Journal of the Science of Food and Agriculture*, 92(7), 1375–1383. https://doi.org/10.1002/jsfa.4709

Samborska, I. A., Kalaji, H. M., Sieczko, L., Goltsev, V., Borucki, W. & Jajoo, A. (2018). Structural and functional disorder in the photosynthetic apparatus of radish plants under magnesium deficiency. *Functional Plant Biology*, 45, 668–679.

Sun, O. J., Gielen, G. J., Sands, R., Smith, T. C., & Thorn, A. J. (2001). Growth, Mg nutrition and photosynthetic activity in *Pinus radiata*: Evidence that NaCl addition counteracts the impact of low Mg supply. *Trees - Structure and Function,* 15(6), 335–340. https://doi.org/10.1007/s004680100111

Sun, O. J., & Payn, T. W. (1999). Magnesium nutrition and photosynthesis in *Pinus radiata*: clonal variation and influence of potassium. *Tree Physiology*, 19(8), 535–540. https://doi.org/10.1093/treephys/19.8.535

Sun, X., Chen, J., Liu, L., Rosanoff, A., Xiong, X., Zhang, Y. & Pei, T. (2018). Effects of magnesium fertilizer on the forage crude protein content depend upon available soil Nitrogen. *Journal of Agricultural and Food Chemistry*, 66, 1743-1750.

Tang, N., Li, Y. & Chen, L. S. (2012). Magnesium deficiency-induced impairment of photosynthesis in leaves of fruiting *Citrus reticulata* trees accompanied by up-regulation of antioxidant metabolism to avoid photo-oxidative damage. *Journal of Plant Nutrition and Soil Science*, 175, 784–793.

Toxopeus, M. R. J. & Gordon, R. B. (1985). Pasture responses to magnesium fertilisers on a yellow-brown pumice soil. *New Zealand Journal of Experimental Agriculture*, 13, 39-45.

Tatagiba, S. D., Damatta, F. M., & Rodrigues, F. A. (2016). Magnesium decreases leaf scald symptoms on rice leaves and preserves their photosynthetic performance. *Plant Physiology and Biochemistry*, 108, 49–56. https://doi.org/10.1016/j.plaphy.2016.07.002

Tränkner, M., Tavakol, E. & Jákli, B. (2018). Functioning of potassium and magnesium in photosynthesis, photosynthate translocation and photoprotection. *Physiologia Plantarum*, 163, 186 414-431.

Troyanos, Y. E., Hipps, N. A., Moorby, J. & Ridout, M. S. (1997). The effects of external magnesium concentration on the growth and magnesium inflow rates of micropropagated cherry rootstocks 'F.12/1' (*Prunus avium* L.) and 'Colt' (*Prunus avium* L. x *Prunus pseudocerasus* L.). *Plant and Soil*, 197, 25–33.

Uzİlday, R. Ö., Uzİlday, B., Yalçinkaya, T., & Türkan, İ. (2017). Mg deficiency changes the isoenzyme pattern of reactive oxygen species-related enzymes and regulates NADPH-oxidase-mediated ROS signalling in cotton. *Turkish Journal of Biology*, 41, 868–880. https://doi.org/10.3906/biy-1704-39

Verbruggen, N., & Hermans, C. (2013). Physiological and molecular responses to magnesium nutritional imbalance in plants. *Plant and Soil*, 368(1–2), 87–99. https://doi.org/10.1007/s11104-013-1589-0

Willson, K. G., Perantoni, A. N., Berry, Z. C., Eicholtz, M. I., Tamukong, Y. B., Yarwood, S. A., & Baldwin, A. H. (2017). Influences of reduced iron and magnesium on growth and photosynthetic performance of *Phragmites australis* subsp. *americanus* (North American common reed). *Aquatic Botany*, 137, 30–38.

Xiao, J. X., Hu, C. Y., Chen, Y. Y., Yang, B., & Hua, J. (2014). Effects of low magnesium and an arbuscular mycorrhizal fungus on the growth, magnesium distribution and photosynthesis of two citrus cultivars. *Scientia Horticulturae*, 177, 14–20. https://doi.org/10.1016/j.scienta.2014.07.016

Yang, G. H., Yang, L. T., Jiang, H. X., Li, Y., Wang, P., & Chen, L. S. (2012). Physiological impacts of magnesium-deficiency in Citrus seedlings: Photosynthesis, antioxidant system and carbohydrates. *Trees - Structure and Function*, 26(4), 1237–1250. https://doi.org/10.1007/s00468-012-0699-2

Yilmaz, O., Kahraman, K., & Ozturk, L. (2017). Elevated carbon dioxide exacerbates adverse effects of Mg deficiency in durum wheat. *Plant and Soil*, 410(1–2), 41–50. https://doi.org/10.1007/s11104-016-2979-x

Ze, Y., Yin, S., Ji, Z., Luo, L., Liu, C. & Hong, F. (2009). Influences of magnesium deficiency and cerium on antioxidant system of spinach chloroplasts. *BioMetals*, 22, 941–949.

Zhang, F., Du, P., Song, C. & Wu, Q. (2015). Alleviation of mycorrhiza to magnesium deficiency in trifoliate orange: Changes in physiological activity. *Emirates Journal of Food and Agriculture*, 27, 1.

Zhou, M., Gong, X., Wang, Y., Liu, C., Hong, M., Wang, L., & Hong, F. (2011). Improvement of cerium of photosynthesis functions of maize under magnesium deficiency. *Biological Trace Element* 220 *Research*, 142(3), 760–772. https://doi.org/10.1007/s12011-010-8769-z
